# Supplementary material for: A panel of nanobodies recognizing conserved hidden clefts of all SARS-CoV-2 spike variants including Omicron
Source: Commun Biol. 2022 Jul 6;5:669. doi: 10.1038/s42003-022-03630-3 (PMC9257560; doi:10.1038/s42003-022-03630-3)
Supplement: Supplementary file 2 — Supplementary Information [file 42003_2022_3630_MOESM2_ESM.pdf]

Supplementary Information for

A panel of nanobodies recognizing conserved hidden clefts of all SARS-CoV-2 spike variants including Omicron

Ryota Maeda*, Junso Fujita, Yoshinobu Konishi, Yasuhiro Kazuma, Hiroyuki Yamazaki, Itsuki Anzai, Tokiko Watanabe, Keishi Yamaguchi, Kazuki Kasai, Kayoko Nagata, Yutaro Yamaoka, Kei Miyakawa, Akihide Ryo, Kotaro Shirakawa, Kei Sato, Fumiaki Makino, Yoshiharu Matsuura, Tsuyoshi Inoue, Akihiro Imura*, Keiichi Namba*, and Akifumi Takaori-Kondo*

*Correspondences:

maeda@cognano.co.jp (R.M.) COGNANO Inc., Kyoto, 601-1255, Japan.

akihiroimura@cognano.co.jp (A.I.) COGNANO Inc., Kyoto, 601-1255, Japan.

keiichi@fbs.osaka-u.ac.jp (Ke.N.) Graduate School of Frontier Biosciences, Osaka University, Osaka 565-0871, Japan.

atakaori@kuhp.kyoto-u.ac.jp (A.T.-K.) Department of Haematology and Oncology, Graduate School of Medicine, Kyoto University, Kyoto 606-8507, Japan.

This PDF file includes:

Supplementary Figures 1 to 11

Supplementary Table 1

Supplementary Figure 1

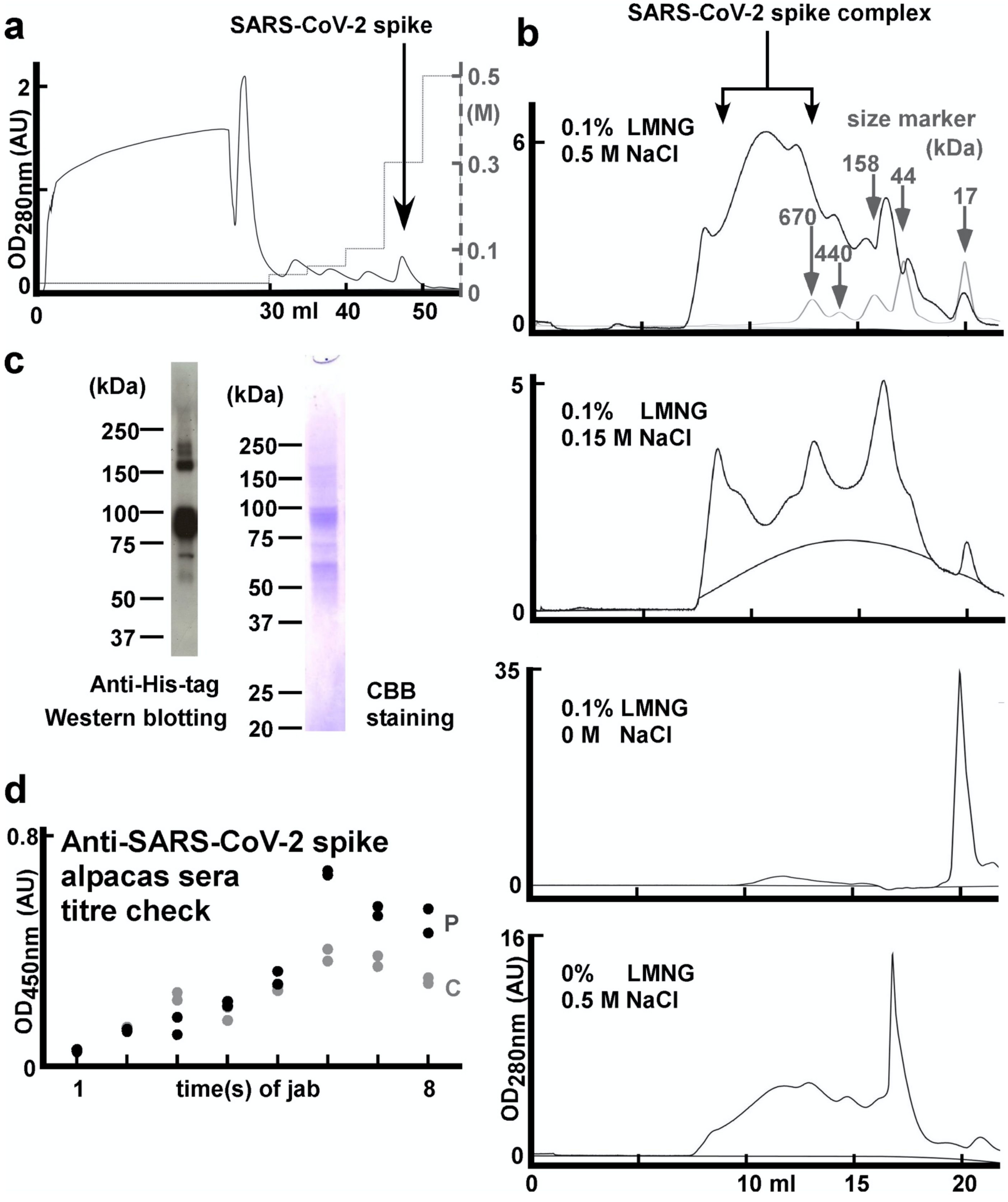

Supplementary Figure 1| Purification of the extracellular SARS-CoV-2 spike complex. **a**, A chromatogram of the first purification step of the SARS-CoV-2 spike from HEK cell lysate using a nickel column: absorbance units (AU) of UV_{280nm}, amounts (ml) of buffer, concentrations (M) of imidazole, and the elution peak containing the SARS-CoV-2 spike. **b**, Chromatograms of the size-exclusion gel filtration of the recombinant SARS-CoV-2 spike complex under different conditions (concentrations of NaCl and LMNG). The first chromatogram is overlayed with those for marker proteins (arrowed). Two arrows indicate the elution peak of the SARS-CoV-2 spike. **c**, Western blotting analysis of the purified SARS-CoV-2 spike using an anti-His antibody and Coomassie Brilliant Blue (CBB) staining. **d**, Serum titres of the immunized alpacas—named P: Puta and C: Christy—for the SARS-CoV-2 spike were measured via ELISA and plotted.

Supplementary Figure 2

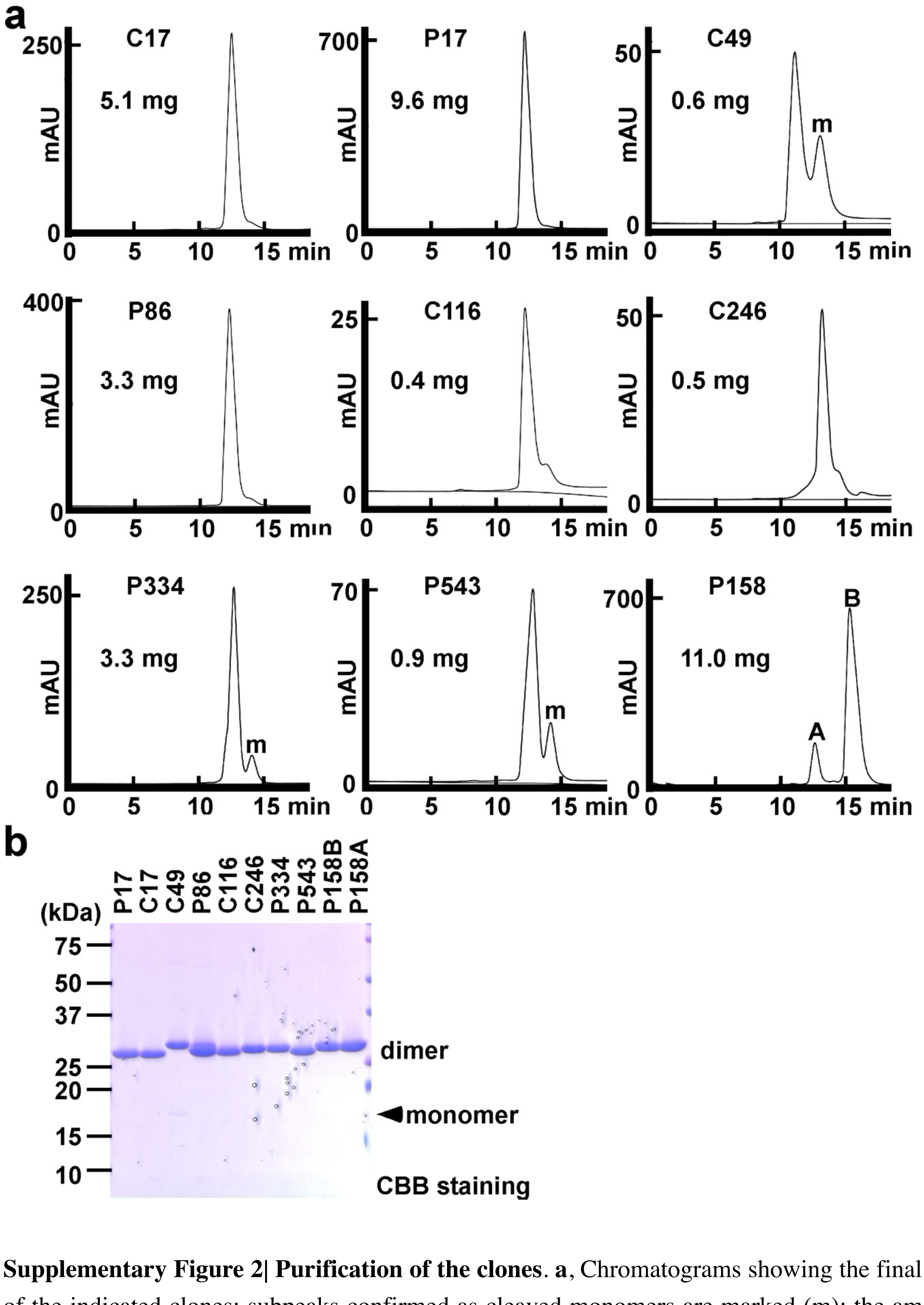

Supplementary Figure 2| Purification of the clones. **a**, Chromatograms showing the final purification step of the indicated clones: subpeaks confirmed as cleaved monomers are marked (m); the amounts of purified clones in dimers from 1 L-culture of BL21 bacteria are shown in insets. The dimer peaks observed for P158 are named A and B. **b**, The purified clones were analysed using CBB staining (m; cleaved monomer). Both the A and B peaks of P158 showed no differences via SDS-PAGE analysis.

Supplementary Figure 3

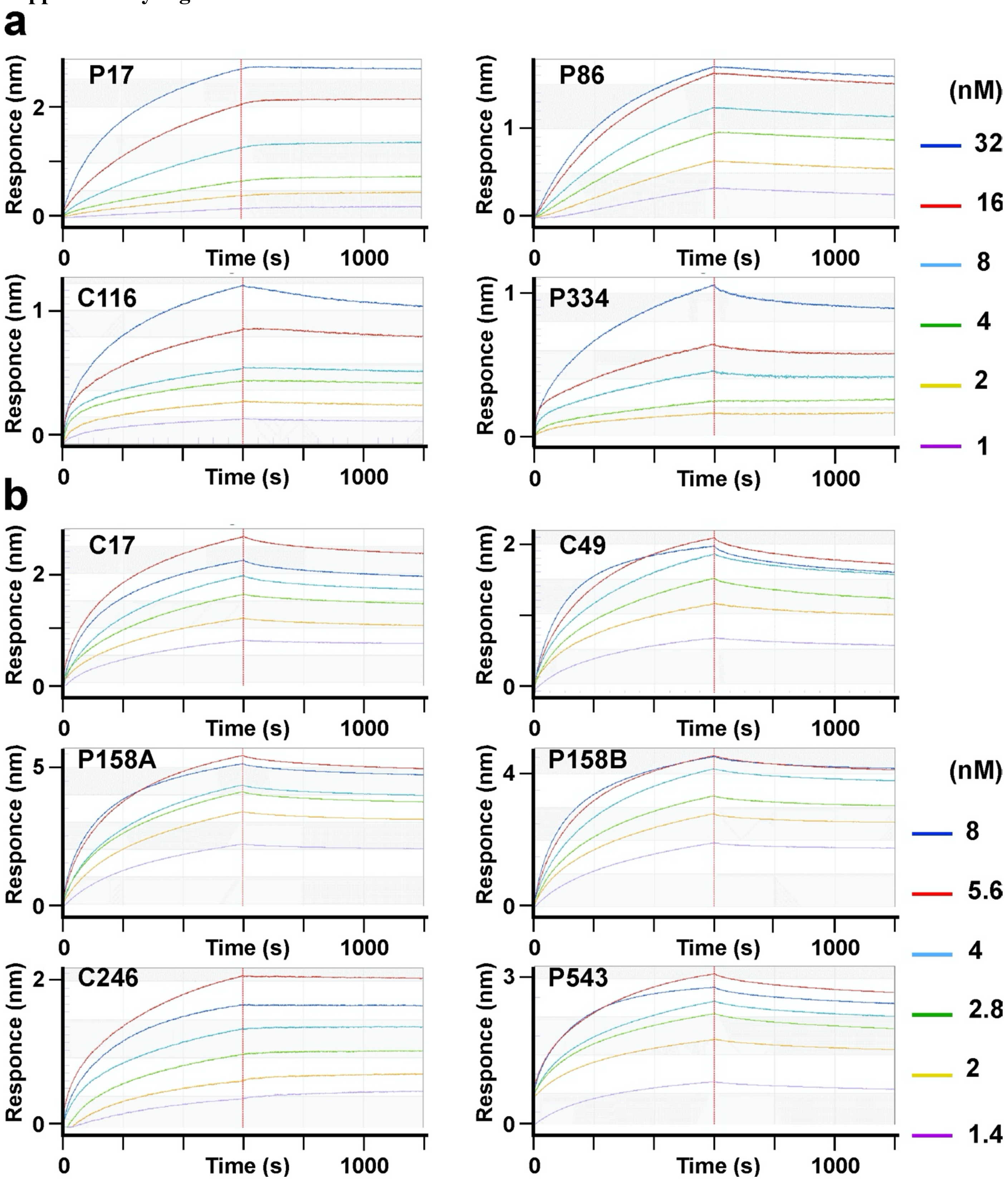

Supplementary Figure 3| Sensorgrams of kinetic assays. **a,b**, Sensorgrams of real-time kinetics assays using biolayer interferometry (BLI)**,** showing five different concentrations of the purified SARS-CoV-2 spike in a high-salt buffer (**a**) or an anionic buffer (**b**).

Supplementary Figure 4

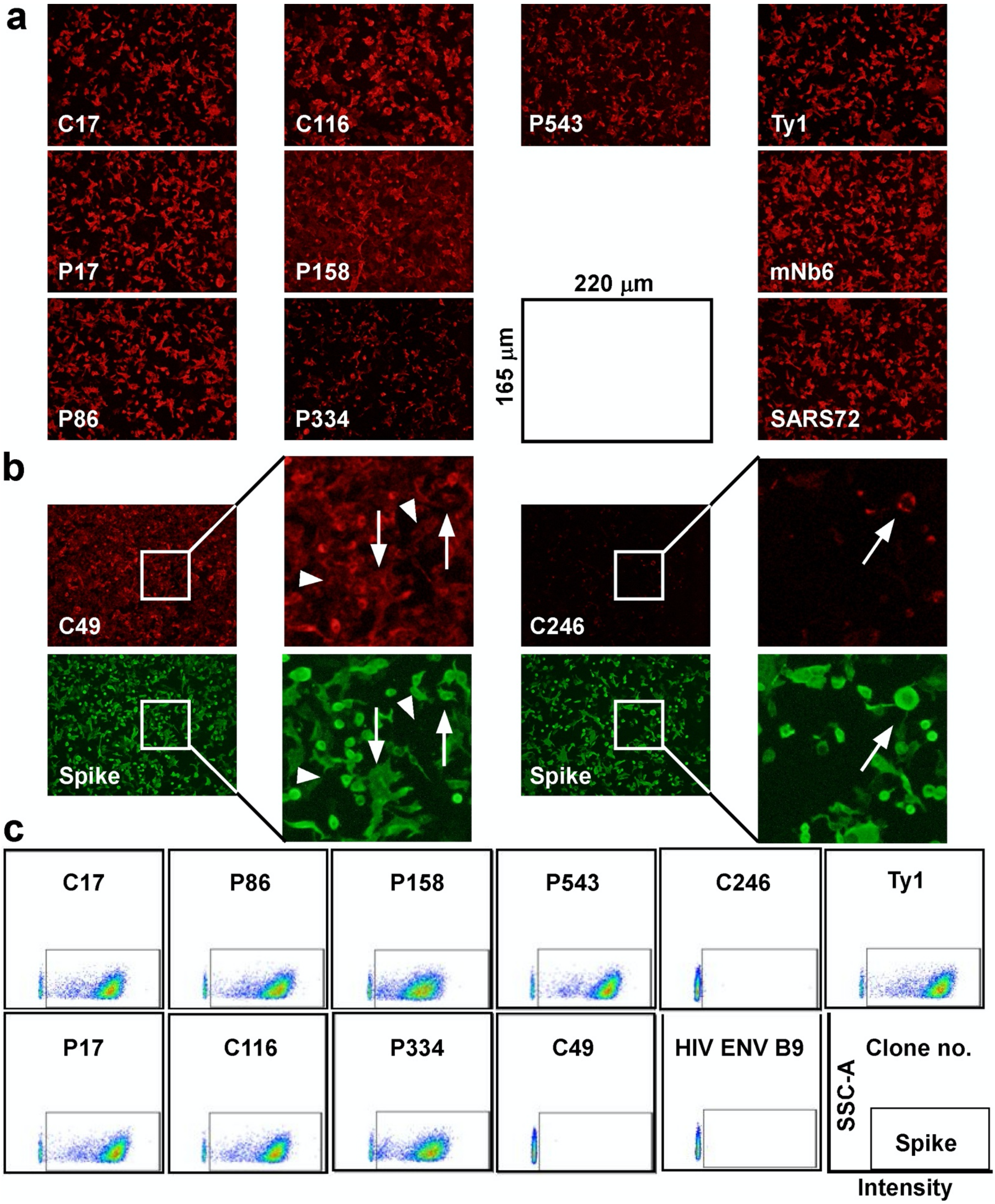

Supplementary Figure 4| Microscopy and flow cytometric analyses of anti-spike nanobodies. a, Photographs of stained HEK293T cells expressing the C9-tagged SARS-CoV-2 spike. **b**, White squared regions show zoomed-in views: arrows indicate intracellularly stained SARS-CoV-2 spike (C49 and C246), and arrowheads indicate example background signals (C49). **c**, Flow cytometric analyses of K562 cells expressing the original SARS-CoV-2 spike—signals with Ty1 squared and B9: an anti-HIV-1 nanobody.

Supplementary Figure 5

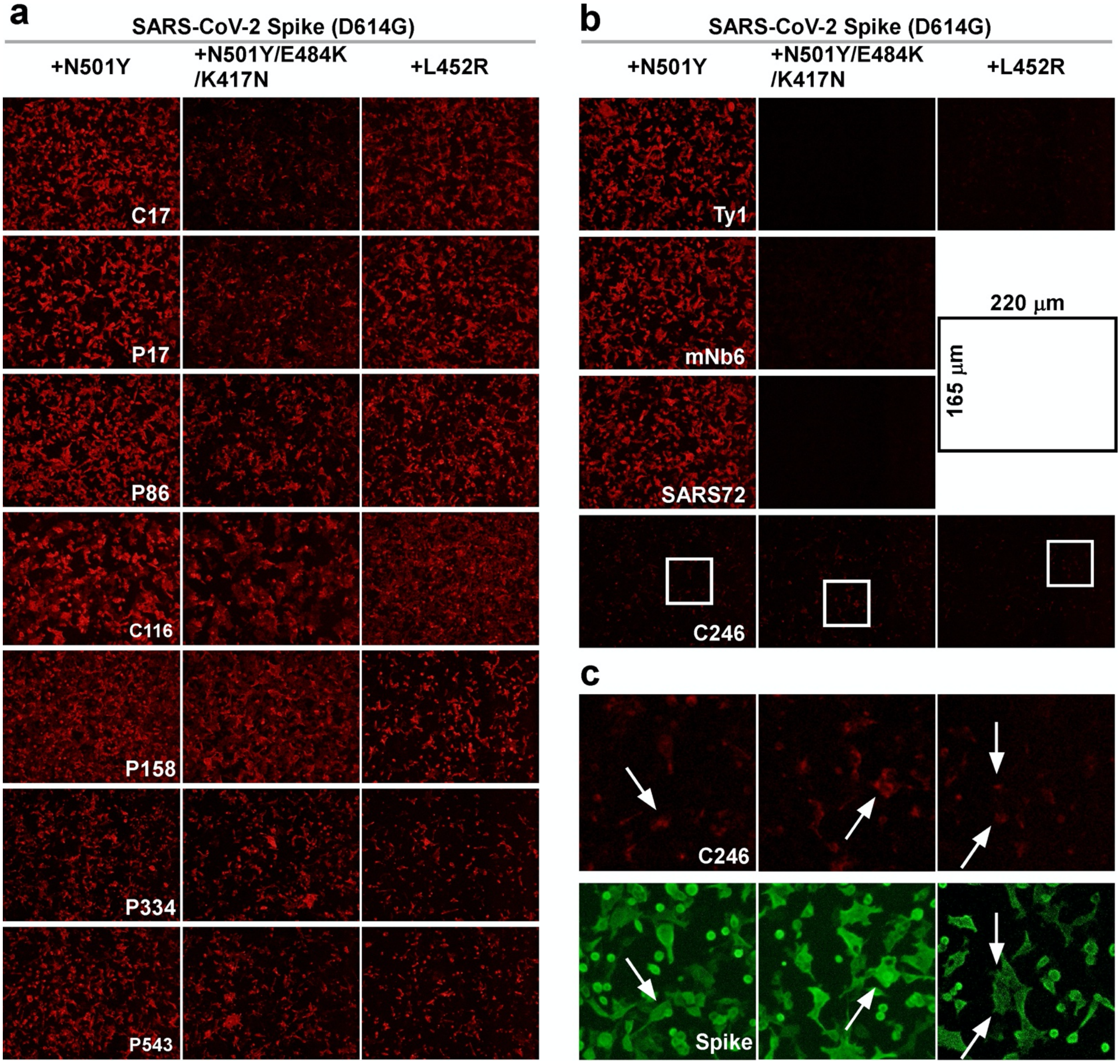

Supplementary Figure 5| Microscopy analyses. a, b, Photographs of stained HEK cells expressing the SARS-CoV-2 spike variants carrying mutations in the RBD, original (D614G); alpha (N501Y); beta (K417N, E484K, and N501Y); and delta (L452R), with generated (**a**) and previously reported (**b**) nanobodies. **c**, Zoomed-in views of regions highlighted with white squares in (**b**): C246 intracellularly stained all spike variants without background—indicated by white arrows.

Supplementary Figure 6

a

P158-coated plate

Blocking	Detection	P17	C17	P158	P334	P543	C116	P86	Ty1
Carbo-Free (CF)	–	0.04	0.07	0.11	0.06	0.06	0.03	0.04	0.05
	+	0.28	0.26	0.31	0.14	0.37	0.11	0.15	0.05
Skim-Milk (Milk)	–	0.02	0.04	0.04	0.03	0.06	0.01	0.04	0.02
	+	0.12	0.11	0.15	0.02	0.29	0.02	0.08	0.03
Albumin (BSA)	–	0.07	0.09	0.26	0.08	0.11	0.07	0.09	0.06
	+	0.70	0.68	0.18	0.15	0.77	0.10	0.33	0.09
Casein	–	0.03	0.06	0.06	0.12	0.07	0.03	0.03	0.01
	+	0.10	0.11	0.27	0.03	0.13	0.04	0.07	0.03
PVP (Polyvinylpyrrolidone)	–	0.14	0.18	0.21	0.12	0.13	0.07	0.10	0.22
	+	0.22	0.24	0.19	0.12	0.26	0.07	0.16	0.14
PBST (0.005%Tween20)	–	0.07	0.20	0.26	0.11	0.26	0.09	0.16	0.11
	+	0.64	0.71	0.31	0.12	0.59	0.06	0.31	0.11

Signal ratio

	P17	C17	P158	P334	P543	C116	P86	Ty1
CF	6.4	3.9	2.9	2.2	6.6	3.6	3.7	0.9
Milk	5.9	2.9	3.7	0.7	5.2	1.5	2.3	1.9
BSA	10.6	8.0	0.7	1.8	6.8	1.4	3.5	1.7
Casein	3.8	2.0	4.7	0.3	1.8	1.2	1.9	2.1
PVP	1.5	1.3	0.9	1.1	2.0	0.9	1.6	0.6
PBST	9.4	3.6	1.2	1.1	2.3	0.6	2.0	0.9

b

Summary of ELISA conditions

Capture: P158	Blocking: CF	Milk	BSA	Casein	PBST
Detection: P543	6.6	5.2	6.8	<2.0	2.3
P86	3.7	2.3	3.5	<2.0	2.0
P17	6.4	5.9	10.6	3.8	9.4
C17	3.9	2.9	8.0	2.0	3.6
P158	2.9	3.7	<2.0	4.7	<2.0

Supplementary Figure 6| ELISA condition screenings. a, P158 was coated on an ELISA plate as a capture antibody; five kinds of blocking solutions (grey)—Carbo-Free (CF), skim milk (Milk), bovine serum albumin (BSA), casein, polyvinylpyrrolidone (PVP), and nonblocking saline (PBST)—were tested. Signals from 2 µg of the purified SARS-CoV-2 spike (+) or blocking buffer only (–) were compared among eight clones used as detection antibodies. Measured optical densities (OD=450 nm) and signal ratios (relative changes in $OD_{450\ nm}$ with (+) compared to without the spike protein (–)) are gradually coloured from green (low) to red (high). **b**, Summary of the top five detection antibodies and appropriate blocking conditions for the P158-based sandwich ELISA: ratios >3.0 are shown in black.

Supplementary Figure 7

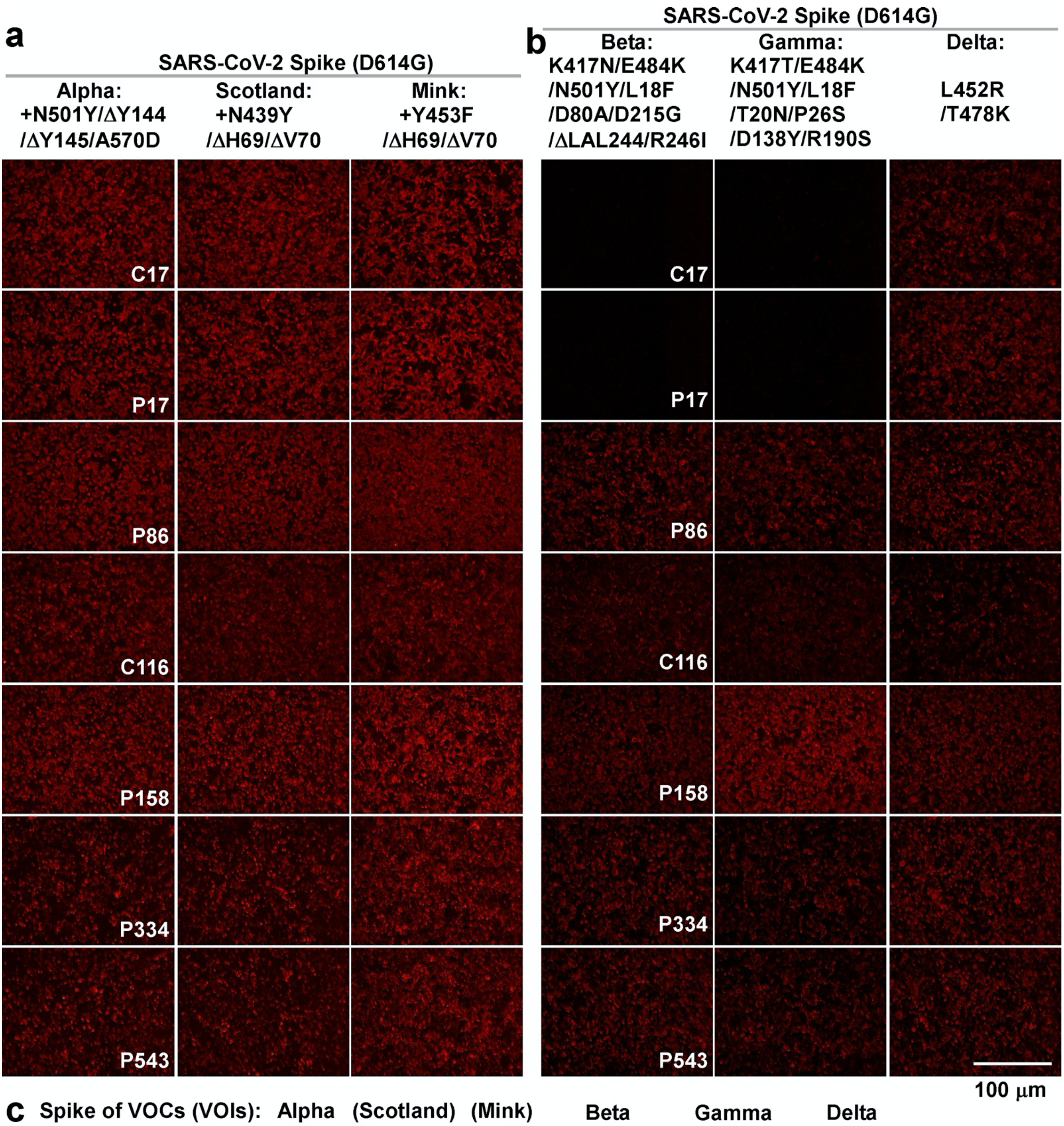

c

Spike of VOCs (VOIs):	Alpha	(Scotland)	(Mink)	Beta	Gamma	Delta
C17	+	+	+	−	−	+
P17	+	+	+	−	−	+
P86	+	+	+	+	+	+
C116	+	+	+	+−	+−	+−
P158	+	+	+	+	+	+
P334	+	+	+	+	+	+
P543	+	+	+	+	+	+

Supplementary Figure 7| Microscopy analyses. Photographs of stained HEK cells expressing the SARS-CoV-2 spike (D614G) variants carrying mutations in the S1 domain: **a, b**, alpha (N501Y, delY144, delY145, and A570D); Scotland (N439Y, delV69, and delH70); Danish mink (Y453F, delV69, and delH70); beta (K417N, E484K, N501Y, L18F, D80A, D215G, delL242, delA243, delL244, and R246I); gamma (K417T, E484K, N501Y, L18F, T20N, P26S, D138Y, and R190S); and delta (L452R and T478K). **c**, Results of microscopy analyses are summarized: +) positive; +–) weak; or –) negative. The SARS-CoV-2 spike variants of interest (VOIs)—Scotland and Danish mink—are shown in parentheses.

Supplementary Figure 8

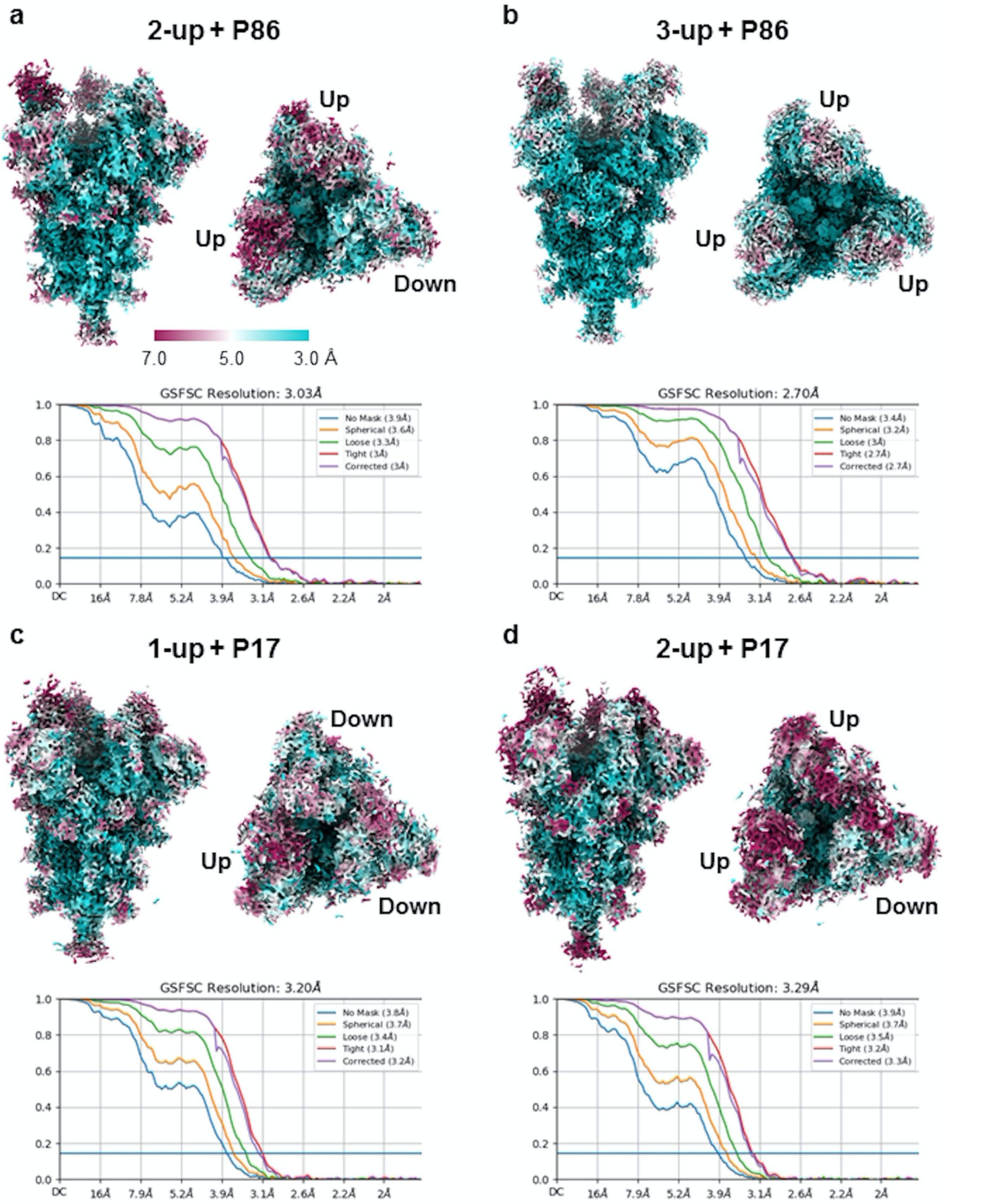

Supplementary Figure 8| Cryo-EM density maps of spike-nanobody complexes SARS-CoV-2 spike-P17 complexes. a-d, Upper panels show final sharpened maps of 2-up+P86 (**a**), 3-up+P86 (**b**), 1-up+P17 (**c**), and 2-up+P17 (**d**) datasets from side views (left) and top views (right) coloured by local resolutions (3.0-7.0 Å). Lower panels show the Fourier shell correlation (FSC) curves for the corresponding final maps of the datasets.

Supplementary Figure 9

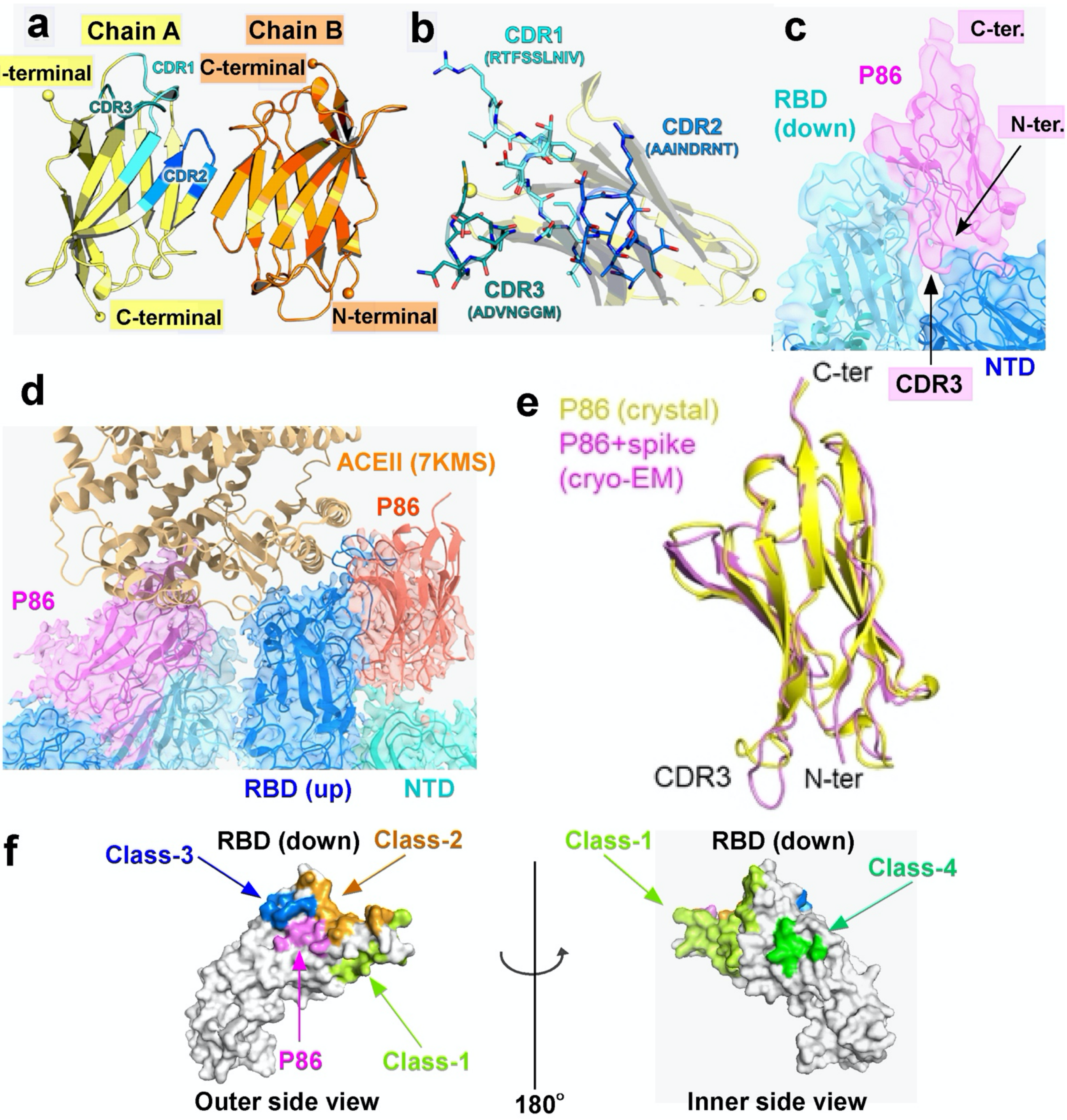

Supplementary Figure 9| Crystal structure of P86. a, Two P86 monomers in the asymmetric unit. Chain A and Chain B are almost identical, with a root mean square deviation for C_α atoms of 0.20 Å. **b**, Close-up view of three CDR regions in Chain A. **c,** Sharpened map from local refinement (5.13 Å resolution at FSC=0.143) around P86 bound to the down-RBD with our fitted model. **d,** Close-up view around P86 bound to the up-RBD with our fitted model structure. The model of ACEII bound to the spike trimer (PDB entry: 7KMS) is superposed on the up-RBD shown in blue. **e,** Structure comparison between P86 alone (crystal structure) and P86 complexed with spike trimer (cryo-EM). **f**, Epitope mapping on the down-RBD. The epitopes of Class-1 to Class-4 compared to P86.

Supplementary Figure 10

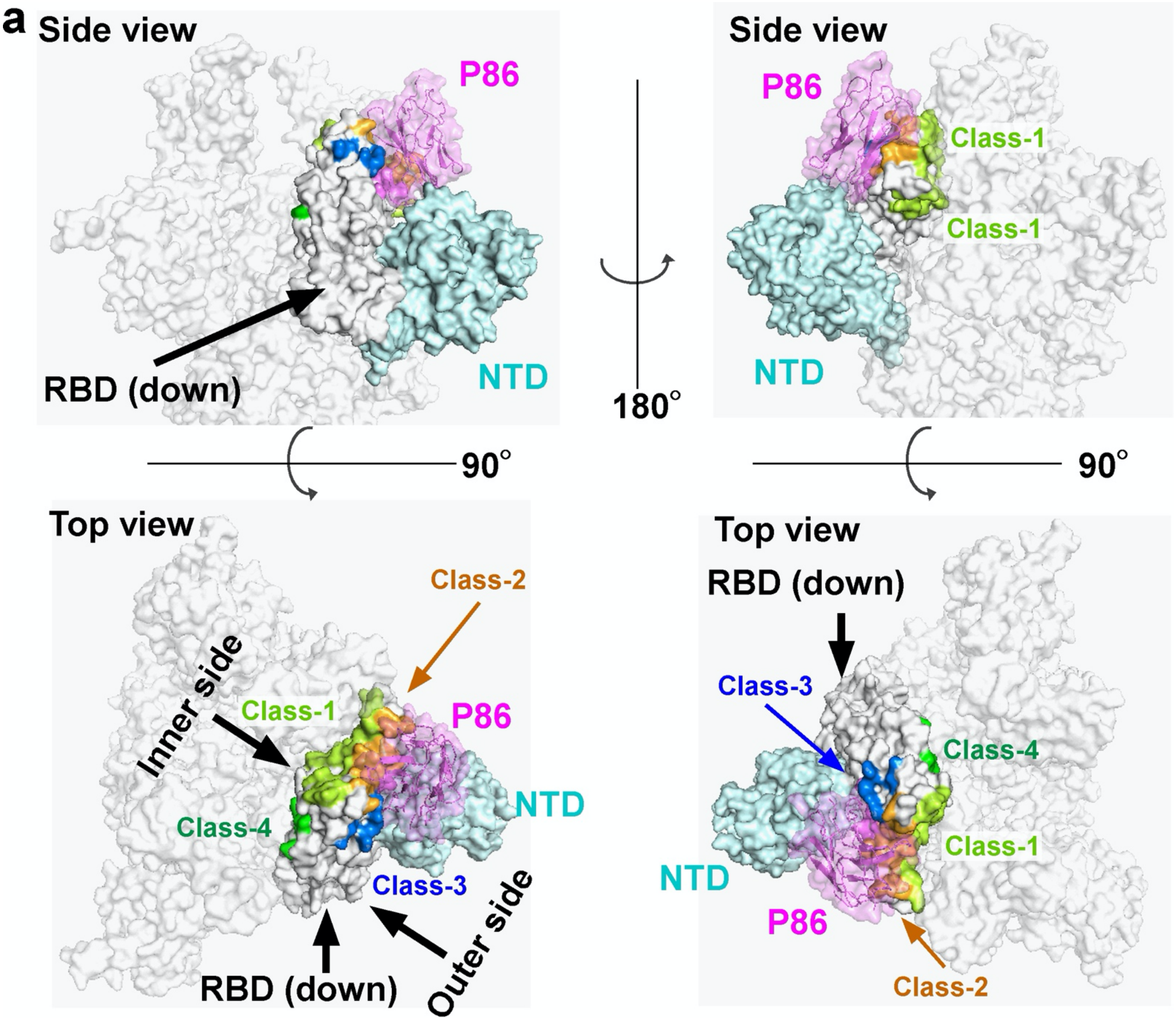

Supplementary Figure 10| The fitting model of P86 and the SARS-CoV-2 spike trimer. a, The down-RBD, the neighbouring NTD, and P86 are coloured white, cyan, and pink, respectively; other domains and molecules of the SARS-CoV-2 spike trimer are in reduced transparency.

Supplementary Figure 11

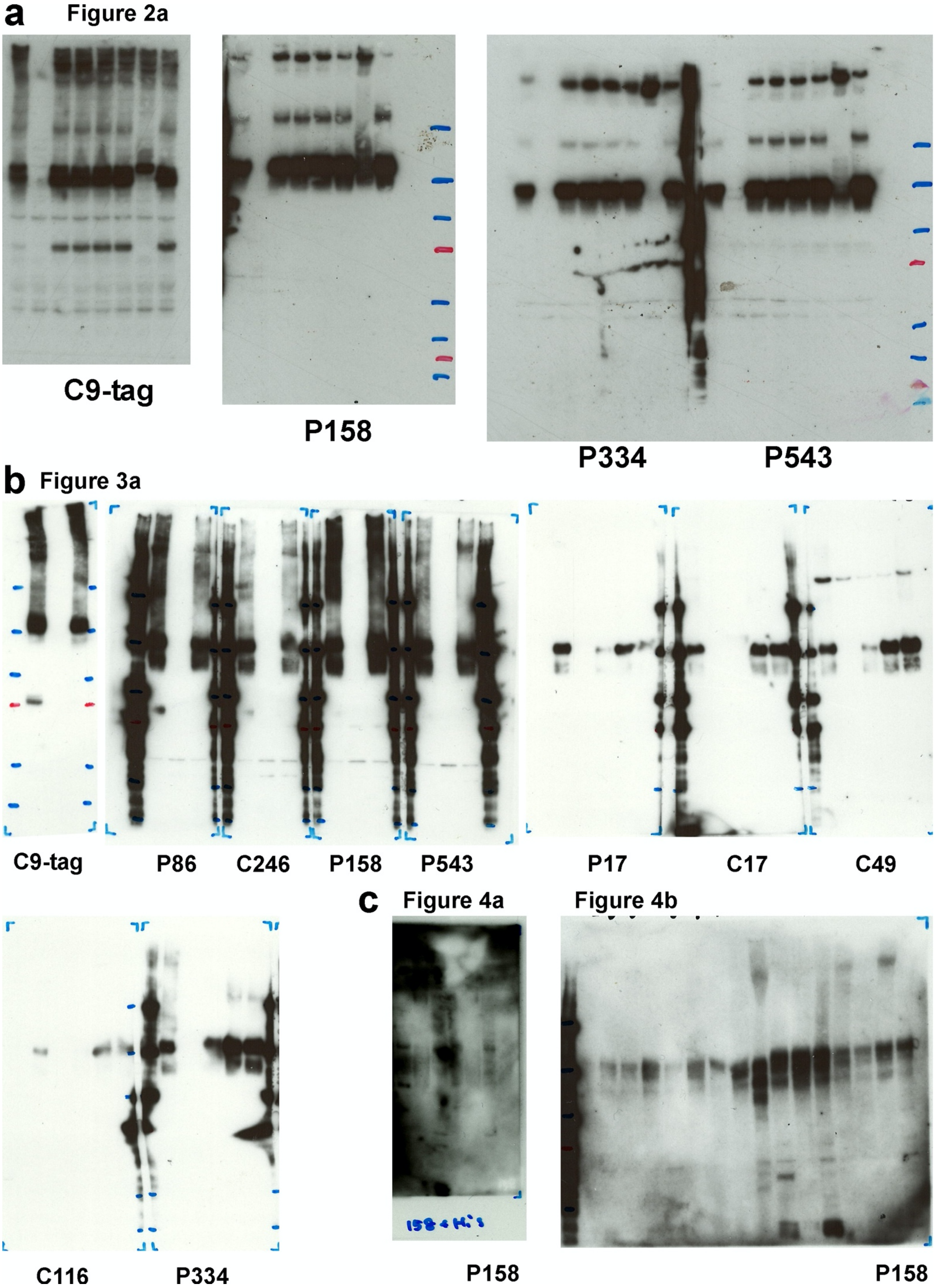

Supplementary Figure 11| Original Western blot images.

Supplementary Table 1| Comparison of the epitopes of Up-RBD+P86 via Protein Interface, Surfaces, and Assemblies (PISA).

No. residues		Accessible Surface Area (Up)	Buried Surface Area and Bonds (H, S)* (Up)	Solvation Energy Effect: ΔG (Up)	Accessible Surface Area (Down)	Buried Surface Area and Bonds (H, S)* (Down)	Solvation Energy Effect: ΔG (Down)
BA.1+BA.2		Å²	Å²	kcal mol⁻¹	Å²	Å²	kcal mol⁻¹
344	Ala	25.1	0	0	31.53	0	0
345	Thr	110.91	0	0	124.78	0	0
346	Arg	173.84	77.26 (S)	–1.54	192.26	72.1	–0.75
347	Phe	21.45	9.37	–0.08	10.98	5.73	–0.02
348	Ala	28.97	26.55	0.37	15.75	15.75	0.25
349	Ser	11.77	9.43 (H)	–0.08	3.61	3.61	0.25
350	Val	0	0	0	0	0	0
351	Tyr	74.5	62.49	–0.03	78.1	70.74 (H)	0.27
352	Ala	48.52	48.02 (H)	0.57	9.91	5.39	0.03
353	Trp	30.81	26.59	0.08	9.86	0.49	–0.01
354	Asn	64	32.34 (H)	–0.23	60.18	27.1	–0.05
355	Arg	103.72	18.72	0.14	71.68	5.51	–0.06
356	Lys	51.9	0	0	91.92	0	0
357	Arg	144.31	0	0	141.1	0	0
445	Val	154.15	0	0	137.36	0	0
446	Gly	20.66	0	0	11.42	0	0
447	Gly	26.79	8.7	0.14	32.48	0	0
448	Asn	3.92	3.92	–0.04	95.95	36.09 (H)	0.13
449	Tyr	86.46	29.68	0.38	45.61	7.23	0
450	Asn	20.7	13.06	–0.15	33.66	25.14	–0.26
451	Tyr	116.94	76.62	0.96	47.31	39.99 (H)	0.56
452	Leu	43.32	37.5	0.6	64.98	62.3 (H)	1
453	Tyr	39.66	0	0	36.07	0	0
454	Arg	21.47	0	0	4.62	0	0
464	Phe	80.55	0	0	50.92	0	0
465	Glu	59.93	0	0	42.78	0	0
466	Arg	3.58	0.44	–0.01	155.78	86.19 (H, S)	–1.91
467	Asp	123.2	23.66	0.02	38.29	0	0
468	Ile	79.87	47.65	0.38	69.49	43.98	0.7
469	Ser	58.67	1.34	0.02	57.58	0	0
470	Thr	47.03	37.4	0.09	49.99	39.84	0.36
471	Glu	117.88	65.91 (H)	0.15	140.23	104.47 (H)	–0.62

472	Ile	32.26	1	0.02	34.84	0	0
473	Tyr	51.04	0	0	20.84	0	0
474	Gln	71.55	0	0	13.1	0	0
475	Ala	32.8	0	0	30.71	0	0
476	Gly	4.57	0	0	62.89	0	0
477	Ser	95.29	0	0	86.67	0	0
478	Thr	123.99	0	0	34.24	0	0
479	Pro	65.07	0	0	82.4	0	0
480	Cys	101.19	0	0	95.47	6.5 (H)	–0.07
481	Asn	122.21	46.78 (H)	–0.54	95.46	1.67	0.03
482	Gly	47.69	35.42 (H)	–0.07	80.11	59.16	–0.3
483	Val	117.5	103.34 (H)	1.39	45.42	31.64 (H)	0.08
484	Glu	11.41	5.08	–0.05	101.28	29.07 (H)	0.02
485	Gly	70.05	37.63 (H)	0.16	0	0	0
486	Phe	116.09	0	0	0	0	0
487	Asn	84.63	0	0	0	0	0
488	Cys	56.1	29.74	0.13	38.53	5.76	0.24
489	Tyr	179.1	3.17	0.05	0	0	0
490	Phe	11.21	6.25	0.05	92.52	72.76	1.16
491	Pro	13.66	0	0	0	0	0
492	Leu	88.49	79.6	1.13	53.01	43.04	0.41
493	Gln	98.13	75.32 (H)	0.31	60.1	19.21	–0.08
494	Ser	96.92	6.52	0.1	74.39	37.03	0.14
495	Tyr	13.89	13.89	–0.15	24.32	11.03	–0.13
496	Gly	30.17	12.9	0.21	28.5	3.84	0.06
497	Phe	14.23	13.45 (H)	–0.15	12.94	12.94 (H)	0
498	Gln	93.16	37.94	0.06	75.68	24.12	–0.24
499	Pro	71.07	2.68	0.04	0	0	0
500	Thr	104.74	0	0	0	0	0
501	Asn	18.17	0	0	0	0	0
502	Gly	47.26	0	0	0	0	0
108	Thr				0	0	0
109	Thr				142.27	8.75	0.06
129	Lys				0	0	0
130	Val				56.73	13.39	0.21
158	Arg				175.29	0	0
159	Val				18.68	0	0
160	Tyr				94.9	22.88	0.37
161	Ser				97.2	4.17	–0.02
162	Ser				165.42	106.89 (H)	0.2
230	Pro				94.51	0	0

231	Ile				18.32	2.87	–0.03
232	Gly				63.8	11.33 (H)	–0.13
233	Ile				54.69	9.66	0.15
234	Asn				104.25	0	0

Bonds*: hydrogen bond (H) and salt bridge (S)

The mutations seen in the BA.1 and the BA.2 lineages are underlined.

The R346K mutation is seen in the BA.1^{+} lineages.

Residues 108 to 234 are in the NTD.
