# Supplementary material for: A panel of nanobodies recognizing conserved hidden clefts of all SARS-CoV-2 spike variants including Omicron
Source: Commun Biol. 2022 Jul 6;5:669. doi: 10.1038/s42003-022-03630-3 (PMC9257560; doi:10.1038/s42003-022-03630-3)
Supplement: Supplementary file 3 — Description of Additional Supplementary Files [file 42003_2022_3630_MOESM3_ESM.pdf]

# Description of Additional Supplementary Files

**File name:** Supplementary Data 1
**Description:** The source data behind the graphs in the paper
