# Supplementary material for: A panel of nanobodies recognizing conserved hidden clefts of all SARS-CoV-2 spike variants including Omicron
Source: Commun Biol. 2022 Jul 6;5:669. doi: 10.1038/s42003-022-03630-3 (PMC9257560; doi:10.1038/s42003-022-03630-3)
Supplement: Supplementary file 5 — Reporting summary [file 42003_2022_3630_MOESM5_ESM.pdf]

nature portfolio

Corresponding author(s): Akihiro Imura, Keiichi Namba, and Akifumi Takaori-Kondo

Last updated by author(s): Jun 7, 2022

Reporting Summary

Nature Portfolio wishes to improve the reproducibility of the work that we publish. This form provides structure for consistency and transparency in reporting. For further information on Nature Portfolio policies, see our Editorial Policies and the Editorial Policy Checklist.

Statistics

For all statistical analyses, confirm that the following items are present in the figure legend, table legend, main text, or Methods section.

n/a	Confirmed	
☐	☒	The exact sample size (*n*) for each experimental group/condition, given as a discrete number and unit of measurement
☐	☒	A statement on whether measurements were taken from distinct samples or whether the same sample was measured repeatedly
☐	☒	The statistical test(s) used AND whether they are one- or two-sided<br>*Only common tests should be described solely by name; describe more complex techniques in the Methods section.*
☒	☐	A description of all covariates tested
☒	☐	A description of any assumptions or corrections, such as tests of normality and adjustment for multiple comparisons
☐	☒	A full description of the statistical parameters including central tendency (e.g. means) or other basic estimates (e.g. regression coefficient) AND variation (e.g. standard deviation) or associated estimates of uncertainty (e.g. confidence intervals)
☐	☒	For null hypothesis testing, the test statistic (e.g. *F*, *t*, *r*) with confidence intervals, effect sizes, degrees of freedom and *P* value noted<br>*Give P values as exact values whenever suitable.*
☒	☐	For Bayesian analysis, information on the choice of priors and Markov chain Monte Carlo settings
☒	☐	For hierarchical and complex designs, identification of the appropriate level for tests and full reporting of outcomes
☒	☐	Estimates of effect sizes (e.g. Cohen's *d*, Pearson's *r*), indicating how they were calculated

Our web collection on statistics for biologists contains articles on many of the points above.

Software and code

Policy information about availability of computer code

Data collection	UNICORN v7.0, Octet Data Acquisition v9.0, Cytomics FC500 CXP, FACSuite v1.2.1, Microplate Manager v6, Glomax Navigator v3, ARVO X3 WorkOut v2.5, MiSeq Reagent Kit v3, cellSens Standard v1.11, SerialEM v3.8, JAFIS v2
Data analysis	UNICORN v7.0, Octet Data Analysis v11.1, Canvas v15, Excel v16.55, Cytomics FC500 CXP, FACSuite v1.2.1, cutadapt v1.18, Trimmomatic v0.39, fastq-join, EMBOSS v6.6.0.0, seqkit v0.10.1, usearch v11, RELION v3.1.1, MotionCor2 v1.4.0, CTFFIND v4.1.14, cryoSPARC v3.2.0, UCSF Chimera v1.19.1, ChimeraX v1.1, MolProbity, PyMOL v2.5.0, XDS, Aimless, MOLREP, PHENIX, Coot, refmac5

For manuscripts utilizing custom algorithms or software that are central to the research but not yet described in published literature, software must be made available to editors and reviewers. We strongly encourage code deposition in a community repository (e.g. GitHub). See the Nature Portfolio guidelines for submitting code & software for further information.

Data

Policy information about availability of data

All manuscripts must include a data availability statement. This statement should provide the following information, where applicable:
- Accession codes, unique identifiers, or web links for publicly available datasets
- A description of any restrictions on data availability
- For clinical datasets or third party data, please ensure that the statement adheres to our policy

Structural density maps and models have been deposited in the Electron Microscopy Data Bank and the Protein Data Bank, and accession codes are described in the methods section. All data are available within the paper and its Supplementary information, or from the corresponding authors upon request.

March 2021

Field-specific reporting

Please select the one below that is the best fit for your research. If you are not sure, read the appropriate sections before making your selection.

☒ Life sciences ☐ Behavioural & social sciences ☐ Ecological, evolutionary & environmental sciences

For a reference copy of the document with all sections, see nature.com/documents/nr-reporting-summary-flat.pdf

Life sciences study design

All studies must disclose on these points even when the disclosure is negative.

Sample size	We collected nasal swab specimens from emergency fever patients (n=22) in the hospital. We believe the sample size is sufficient to obtain relevant analyses. In the cryoEM image processing, we disclosed the sample size in this article and its Supplementary Information files. Totally 878,975 and 766,652 particle images were automatically picked from 4,175 and 2,124 micrographs in the 2-up/3-up+P86 and 1-up/2-up+P17 datasets, respectively. Among these, 38,004, 44,292, 67,529, and 48,715 particle images in the 2-up+P86, 3-up+P86, 1-up+P17, and 2-up+P17 datasets, respectively, were used for final reconstruction because they were classified into higher resolution datasets after 3D classification.
Data exclusions	In the cryoEM image processing by 2D and 3D classification and selection, 796,679 and 650,408 particle images were excluded from the initial 878,975 and 766,652 particle images in the 2-up/3-up+P86 and 1-up/2-up+P17 datasets, respectively.
Replication	Experiments that were repeated are noted in Figure legends. All attempts at replication were successful for structural analysis.
Randomization	Randomization was not applicable and all available samples were tested in this paper.
Blinding	Investigators performed neutralization assays under blinded conditions.

Reporting for specific materials, systems and methods

We require information from authors about some types of materials, experimental systems and methods used in many studies. Here, indicate whether each material, system or method listed is relevant to your study. If you are not sure if a list item applies to your research, read the appropriate section before selecting a response.

Materials & experimental systems

n/a	Involved in the study
☐	☒ Antibodies
☐	☒ Eukaryotic cell lines
☒	☐ Palaeontology and archaeology
☐	☒ Animals and other organisms
☐	☒ Human research participants
☒	☐ Clinical data
☒	☐ Dual use research of concern

Methods

n/a	Involved in the study
☒	☐ ChIP-seq
☐	☒ Flow cytometry
☒	☐ MRI-based neuroimaging

Antibodies

Antibodies used	All antibodies and nanobodies used were described in this manuscript.
Validation	All primary antibodies were noted their supplier and validated on the websites; nanoboies were referred to their original paper. Anti-IL-6 and anti-Her2 nanobodies were validated via biolayer interferometry.

Eukaryotic cell lines

Policy information about cell lines

Cell line source(s)	HEK293T, K562, and HOC cells were from ATCC; Expi293F cells were from Life technologies; DT40 and VeroE6-TMPRSS2 were obtained from the Japanese Collection of Research Bioresources (JCRB) Cell Bank.
Authentication	None cell lines used were authenticated.
Mycoplasma contamination	K562, DT40, HOC, and VeroE6-TMPRSS2 cells had been checked for mycoplasma contamination and always tested negative.
Commonly misidentified lines (See ICLAC register)	No commonly misidentified cell lines were used in this study.

Animals and other organisms

Policy information about studies involving animals; ARRIVE guidelines recommended for reporting animal research

Laboratory animals	Two heads of alpacas (1.5-2 years old, male and female) were maintained at KYODOKEN Institute.
Wild animals	n/a
Field-collected samples	n/a
Ethics oversight	The KYODOKEN Institute Animal Care and Use Committee approved the protocols for this study (approval number 20200312).

Note that full information on the approval of the study protocol must also be provided in the manuscript.

Human research participants

Policy information about studies involving human research participants

Population characteristics	We obtained nasal swab specimens from patients of ages 20-80 with an approximately equal distribution of males and females in Japan.
Recruitment	We recruited patients who had fever in the hospital. Patients were included without any selection.
Ethics oversight	The Committee of Shizuoka City Hospital approved the protocols for this study (approval number 20220128).

Note that full information on the approval of the study protocol must also be provided in the manuscript.

Flow Cytometry

Plots

Confirm that:

☒ The axis labels state the marker and fluorochrome used (e.g. CD4-FITC).

☒ The axis scales are clearly visible. Include numbers along axes only for bottom left plot of group (a 'group' is an analysis of identical markers).

☒ All plots are contour plots with outliers or pseudocolor plots.

☒ A numerical value for number of cells or percentage (with statistics) is provided.

Methodology

Sample preparation	K562 and DT40 cells were freshly prepared and stained as described in the method section.
Instrument	Beckman Caulter Cytomics FC-500 and Becton Dickinson FACSLyric
Software	Cytomics FC-500 CXP software and FACSuite v1.2.1.
Cell population abundance	At least 10,000 cells were acquired for each assay condition.
Gating strategy	Gates were set on B9-negative and Ty1-positive area (B9 for HIV-1 Enverope and Ty1 for SARS-CoV2 spike).

☒ Tick this box to confirm that a figure exemplifying the gating strategy is provided in the Supplementary Information.
